# Nurses’ perceptions of how their professional autonomy influences the moral dimension of end-of-life care to nursing home residents– a qualitative study

**DOI:** 10.1186/s12912-024-01865-5

**Published:** 2024-03-28

**Authors:** Rachel Gilbert, Daniela Lillekroken

**Affiliations:** Department of Nursing and Health Promotion, Oslo Metropolitan University, PB 4, St. Olavs Plass, N-0130 Oslo, Norway

**Keywords:** End-of-life, Moral qualities, Moral dimension, Nurses, Nursing homes, Professional autonomy, Qualitative study

## Abstract

**Background:**

Over the years, caring has been explained in various ways, thus presenting various meanings to different people. Caring is central to nursing discipline and care ethics have always had an important place in nursing ethics discussions. In the literature, Joan Tronto’s theory of ethics of care is mostly discussed at the personal level, but there are still a few studies that address its influence on caring within the nursing context, especially during the provision of end-of-life care. This study aims to explore nurses’ perceptions of how their professional autonomy influences the moral dimension of end-of-life care provided to nursing home residents.

**Methods:**

This study has a qualitative descriptive design. Data were collected by conducting five individual interviews and one focus group during a seven-month period between April 2022 and September 2022. Nine nurses employed at four Norwegian nursing homes were the participants in this study. Data were analysed by employing a qualitative deductive content analysis method.

**Results:**

The content analysis generated five categories that were labelled similar to Tronto’s five phases of the care process: (i) caring about, (ii) caring for, (iii) care giving, (iv) care receiving and (v) caring with. The findings revealed that nurses’ autonomy more or less influences the decision-making care process at all five phases, demonstrating that the Tronto’s theory contributes to greater reflectiveness around what may constitute ‘good’ end-of-life care.

**Conclusions:**

Tronto’s care ethics is useful for understanding end-of-life care practice in nursing homes. Tronto’s care ethics provides a framework for an in-depth analysis of the asymmetric relationships that may or may not exist between nurses and nursing home residents and their next-of-kin. This can help nurses see and understand the moral dimension of end-of-life care provided to nursing home residents during their final days. Moreover, it helps handle moral responsibility around end-of-life care issues, providing a more complex picture of what ‘good’ end-of-life care should be.

## Background

In recent decades, improving end-of-life care has become a global priority [1]. The proportion of older residents dying in nursing homes is rising across the world [2], resulting in a significant need to improve the quality of end-of-life care provided to residents. Therefore, throughout the world, nursing homes are becoming increasingly important as end-of-life care facilities [3]. As the largest professional group in healthcare [4], nurses primarily engage in direct care activities [5] and patient communication [6] positioning them in close proximity to patients. This proximity affords them the opportunity to serve as information brokers and mediators in end-of-life decision-making [7]. They also develop trusting relationships with residents and their next-of-kin, relationships that may be beneficial for the assessment of residents and their next-of-kin’s needs [8]. Moreover, nurses have the opportunity to gain a unique perspective that allows them to become aware of if and when a resident is not responding to a treatment [9].

When caring for residents in their critical end-of-life stage, nurses form a direct and intense bond with the resident’s next-of-kin, hence nurses become central to end-of-life care provision and decision-making in nursing homes [10]. The degree of residents and their next-of-kin involvement in the decision-making process in practice remains a question [11]. Results from a study conducted in six European countries [12], demonstrate that, in long-term care facilities, too many care providers are often involved, resulting in difficulties in reaching a consensus in care. Although nurses believe that their involvement is beneficial to residents and families, there is a need for more empirical evidence of these benefits at the end-of-life stage. However, the question of who should be responsible for making decisions is still difficult to answer [13]. One study exploring nurse’s involvement in end-of-life decisions revealed that nurses experience ethical problems and uncertainty about the end-of-life care needs of residents [14]. Another study [10] reported patients being hesitant to discuss end-of‐life issues with their next-of-kin, resulting in nurses taking over; thus, discussing end-of-life issues became their responsibility. A study conducted in several nursing homes from the UK demonstrated that ethical issues associated with palliative care occurred most frequently during decision-making, causing greater distress among care providers [15].

Previous research has revealed that there are some conflicts over end-of-life care that consume nurses’ time and attention at the resident’s end-of-life period [16]. The findings from a meta-synthesis presenting nurses’ perspectives dealing with ethical dilemmas and ethical problems in end-of-life care revealed that nurses are deeply involved with patients as human beings and display an inner responsibility to fight for their best interests and wishes in end-of-life care [17].

Within the Norwegian context, several studies have explored nurses’ experiences with ethical dilemmas when providing end-of-life care in nursing homes. One study describing nurses’ ethical dilemmas concerning limitation of life-prolonging treatment suggested that there are several disagreements between the next-of-kin’s wishes and what the resident may want or between the wishes of the next-of-kin and what the staff consider to be right [18]. Another study revealed that nurses provide ‘more of everything’ and ‘are left to dealing with everything on their own’ during the end-of-life care process [19]^(p.13)^. Several studies aiming to explore end-of-life decision-making in nursing homes revealed that nurses experience challenges in protecting the patient’s autonomy regarding issues of life-prolonging treatment, hydration, nutrition and hospitalisation [20–22]. Other studies conducted in the same context have described that nurses perceive ethical problems as a burden and as barriers to decision-making in end-of-life care [8, 23].

Nursing, as a practice, is fundamentally grounded in moral values. The nurse-patient relationship, central to nursing care provision, holds ethical importance and significance. It is crucial to recognise that the context within which nurses practice can both shape and be shaped by nursing’s moral values. These values collectively constitute what can be termed the ethical dimension of nursing [24]. Nursing ethos and practices are rooted in ethical values and principles; therefore, one of the position statements of the International Council of Nurses [25] refers to nurses’ role in providing care to dying patients and their families as an inherent part of the International Classification for Nursing Practice [26] (e.g., dignity, autonomy, privacy and dignified dying). Furthermore, ethical competence is recognised as an essential element of nursing practice [27], and it should be considered from the following viewpoints: ethical decision-making, ethical sensitivity, ethical knowledge and ethical reflection.

The term ‘end-of-life care’ is often used interchangeably with various terms such as terminal care, hospice care, or palliative care. End-of life care is defined as care ‘to assist persons who are facing imminent or distant death to have the best quality of life possible till the end of their life regardless of their medical diagnosis, health conditions, or ages’ [28] ^(p.613)^. From this perspective, professional autonomy is an important feature of nurses’ professionalism [29]. Professional autonomy can be defined based on two elements: independence in decision-making and the ability to use competence, which is underpinned by three themes: shared leadership, professional skills, inter- and intraprofessional collaboration and a healthy work environment [30].

As presented earlier, research studies have reported that nurses experience a range of difficulties or shortcomings during the decision-making process; therefore, autonomous practice is essential for safe and quality care [31]. Moreover, autonomous practice is particularly important for the moral dimension in end-of-life care, where nurses may need to assume more responsibility in the sense of defining and giving support to matters that are at risk of not respecting ethical principles or fulfilling their ethical, legal and professional duties towards the residents they care for.

To the best of the researchers’ knowledge, little is known about nurses’ perceptions of how their professional autonomy influences the moral dimension of end-of-life care provided to nursing home residents; therefore, the aim of this study is to explore nurses’ perceptions of how their professional autonomy influences the moral dimension of end-of-life care provided to nursing home residents.

### Theoretical framework

Joan Tronto is an American political philosopher and one of the most influential care ethicists. Her theory of the ethics of care [32–34] has been chosen as the present study’s theoretical framework. The ethics of care is a feminist-based ethical theory, focusing on caring as a moral attitude and a sensitive and supportive response of the nurse to the situation and circumstances of a vulnerable human being who is in need of help [33–35]. In this sense, nurses’ caring behaviour has the character of a means—helping to reach the goal of nursing practice—which here entails providing competent end-of-life care.

Thinking about the process of care, in her early works [32–34], Tronto proposes four different phases of caring and four elements of care. Although the phases may be interchangeable and often overlap with each other, the elements of care are fundamental to demonstrate caring. The phases of caring involve cognitive, emotional and action strategies.

The first phase of caring is *caring about*, which involves the nurse’s recognition of being in need of care and includes concern, worry about someone or something. In this phase, the element of care is attentiveness, which entails the detection of the patient and/or family need.

The second phase is *caring for*, which implies nurses taking responsibility for the caring process. In this phase, responsibility is the element of care and requires nurses to take responsibility to meet a need that has been identified.

The third phase is *care giving*, which encompasses the actual physical work of providing care and requires direct engagement with care. The element of care in this phase is competence, which involves nurses having the knowledge, skills and values necessary to meet the goals of care.

The fourth phase is *care receiving*, which involves an evaluation of how well the care giving meets the caring needs. In this phase, responsiveness is the element of care and requires the nurse to assess whether the care provided has met the patient/next-of-kin care needs. This phase helps preserve the patient–nurse relationship, which is a distinctive aspect of the ethics of care [36].

In 2013, Tronto [35] updated the ethics of care by adding a fifth phase of caring—*caring with*—which is the common thread weaving among the four phases. When care is responded to through care receiving and new needs are identified, nurses return to the first phase and begin again. The care elements in this phase are trust and solidarity. Within a healthcare context, trust builds as patients and nurses realise that they can rely on each other to participate in their care and care activities. Solidarity occurs when patients, next-of-kin, nurses and others (i.e., ward leaders, institutional management) engage in these processes of care together rather than alone.

To the best of our knowledge, these five phases of caring and their elements of caring have never been interpreted within the context of end-of-life care. The ethics of care framework offers a context-specific way of understanding how nurses’ professional autonomy influences the moral dimension of end-of-life care provided to nursing home residents, revealing similarities with Tronto’s five phases, which has motivated choosing her theory.

## Methods

### Aim of the study

The present study aims to explore nurses’ perceptions of how their professional autonomy influences the moral dimension of end-of-life care provided to nursing home residents.

### Design

The current study has a qualitative descriptive design using five individual interviews and one focus group to explore nurses’ perceptions of how their professional autonomy influences the moral dimension of end-of-life care provided to nursing home residents.

### Setting and participants

The setting for the study was four nursing homes located in different municipalities from the South-Eastern region of Norway. Nursing homes in Norway are usually public assisted living facilities and offer all-inclusive accommodation to dependent individuals on a temporary or permanent basis [37]. The provision of care in the Norwegian nursing homes is regulated by the ‘Regulation of Quality of Care’ [38], aiming to improve nursing home residents’ quality of life by offering quality care that meets residents’ fundamental physiological and psychosocial needs and to support their individual autonomy through the provision of daily nursing care and activities tailored to their specific needs, and, when the time comes, a dignified end-of-life care in safe milieu.

End-of-life care is usually planned and provided by nurses having a post graduate diploma in either palliative nursing or oncology nursing– often holding an expert role, hence ensuring that the provision of end-of-life care meets the quality criteria and the resident’s needs and preferences [39].

To obtain rich information to answer the research question, it was important to involve participants familiar with the topic of study and who had experience working in nursing homes and providing end-of-life care to residents; therefore, a purposive sample was chosen. In this study, a heterogeneous sampling was employed, which involved including participants from different nursing homes with varying lengths of employment and diverse experiences in providing end-of-life care to residents. This approach was chosen to gather data rich in information [40]. Furthermore, when recruiting participants, the first author was guided by Malterud et al.’s [41] pragmatic principle, suggesting that the more ‘information power’ the participants provided, the smaller the sample size needed to be, and vice versa. Therefore, the sample size was not determined by saturation but instead by the number of participants who agreed to participate. However, participants were chosen because they had particular characteristics such as experience and roles which would enable understanding how their professional autonomy influences the moral dimension of end-of-life care provided to nursing home residents.

The inclusion criteria for the participants were as follows: (i) to be a registered nurse, (ii) had a minimum work experience of two years employed at a nursing home, and (iii) had clinical experience with end-of-life/palliative care. To recruit participants, the first author sent a formal application with information about the study to four nursing homes. After approval had been given, the participants were asked and recruited by the leadership from each nursing home. The participants were then contacted by the first author by e-mail and scheduled a time for meeting and conducting the interviews.

Ten nurses from four different nursing homes were invited to participate, but only nine agreed. The participants were all women, aged between 27 and 65 and their work experience ranged from 4 to 21 years. Two participants had specialist education in palliative care, and one was currently engaged in a master’s degree in nursing science. Characteristics of the participants are presented in Table 1:

**Table 1:** Participants’ demographic data

Participant no.	Type of interview	Age (years)	Gender	Work experience as a nurse (years)	Further specialist education	Nursing home
1	Ind. interview	52	F	18	Palliative care	A
2	Ind. interview	42	F	19	None	B
3	Ind. interview	65	F	21	Palliative care	B
4	Ind. interview	51	F	12	None	B
5	Ind. interview	45	F	5	None	C
6	Focus group	34	F	9	None	D
7	Focus group	43	F	15	None	D
8	Focus group	38		10	Master (on going education at the time of interview)	D
9	Focus group	27	F	4	None	D

### Data collection methods

Data were collected through five semistructured individual and one focus group interviews. Both authors conducted the interviews together. The study was carried out between April and September 2022. Due to the insecurity related to the situation caused by the post-SARS-CoV-2 virus pandemic and concerns about potential new social distancing regulations imposed by the Norwegian government, four participants from the same nursing home opted for a focus group interview format. This decision was motivated by a desire to mitigate the potential negative impact that distancing regulations might have on data collection. The interviews were guided by an interview guide developed after reviewing relevant literature on end-of-life care and ethical dilemmas. The development of the interview guide consisted of five phases: (i) identifying the prerequisites for using semi-structured interviews; (ii) retrieving and using previous knowledge; (iii) formulating the preliminary semi-structured interview guide; (iv) pilot testing the interview guide; and (v) presenting the complete semistructured interview guide [42]. The interview guide was developed by both authors prior to the onset of the project and consisted of two demographic questions and eight main open-ended questions. The interview guide underwent initial testing with a colleague employed at the same nursing home as the first author. After the pilot phase in phase four, minor language revisions were made to specific questions to bolster the credibility of the interview process and ensure the collection of comprehensive and accurate data. The same interview guide was used to conduct individual interviews and focus group (Table 2).

**Table 2:** Overview of the questions posed during the interviews

Demographic data	How old are you, and how many years have you been working as a nurse?
	How many years of experience do you have working in a nursing home and providing end-of-life care to nursing home residents?
Opening questions	Can you please describe the most recent ethical dilemma you have encountered in your line of work?
	Can you please describe a situation in which you have used your professional autonomy here in nursing home in an end-of-life care situation that you perceived it as ethically challenging?
Main questions posed during the interview	In your opinion, what is an ethical dilemma/problem associated with end-of life care?
	In your opinion, what are the ethical challenges faced by nurses in administration of end-of-life care to nursing home residents?
	Can you please provide some examples of the ethical challenges faced by nurses when there is a conflict between resident or/and next-of-kin over termination of life-prolonging or active medication by the doctor?
	What are the ethical challenges for nurses associated with the introduction of end-of-life medication (Morphine, Midazolam/Versed, Haloperidol and Glycopyrrolate/Robinul) by the doctor?
	In your opinion, what is the ethical way for nurses to exercise their professional autonomy in the administration of end-of-life care to nursing home residents?
	In your opinion, how do you define moral distress, and can you please, provide an example of a situation when you experienced moral distress?
Follow-up questions	Follow-up questions included how frequently such ethical dilemmas arose; whether the dilemma was resolved, if not and why not; if barriers existed to resolving dilemmas; and what resources the nurses used for resolving ethical dilemmas, for example, the importance of building a trusting relationship with resident and their next-of-kin; how end-of-life ethical competence can be improved in nursing homes, and what type of on-the-job ethical training would improve end-of-life care in nursing homes.

The interviews were all conducted in a quiet room at a nursing home. Each interview lasted between 30 and 60 min and were digitally recorded. The individual interviews were transcribed verbatim by the first author. The focus group interview was transcribed by the second author.

### Ethical perspectives

Prior to the onset of the data collection, ethical approval and permission to conduct the study were sought from the Norwegian Agency for Shared Services in Education and Research (Sikt/Ref. number 360,657) and from each leader of the nursing home. The study was conducted in accordance with the principles of the Declaration of Helsinki of the World Medical Association [43]: informed consent, consequences and confidentiality. The participants received written information about the aim of the study, how the researcher would ensure their confidentiality and, if they chose to withdraw from the study, their withdrawal would not have any negative consequences for their employment at nursing homes. Data were anonymised, and the digital records of the interviews were stored safely on a password-protected personal computer. The transcripts were stored in a locked cabinet in accordance with the existing rules and regulations for research data storage at Oslo Metropolitan University. The participants did not receive any financial or other benefits from participating in the study. Written consent was obtained prior to data collection, but verbal consent was also provided before each interview. None of the participants withdrew from the study.

### Data analysis

The data were analysed by employing a qualitative deductive content analysis, as described by Kyngäs and Kaakinen [44]. Both researchers independently conducted the data analysis manually. The empirical data consisted of 63 pages (34,727 words) of transcripts from both individual and focus group interviews. The deductive content analysis was performed in three steps: (i) preparation, (ii) organisation and (iii) reporting of the results.

During the first step—preparation—each researcher, individually, read the transcripts several times to get an overview of the data and select units of analysis by searching for recurring codes and meanings and to carefully compare the similarities and differences between coded data. These codes were labelled independently by both researchers and placed into an analysis matrix.

During the next step—organisation—the researchers met and discussed and then compared and revised the labels several times until they agreed about the preliminary findings. During the interpretative process towards developing an understanding of the empirical data, the content of the labels referred to nurses’ perceptions about how their professional autonomy influences the moral dimension of end-of-life care provided to nursing home residents, revealing similarities with the five phases of Tronto’s theory of ethics of care [32, 33], thus assigning them to the five phases of the theory. Following this final refinement, one main category and five categories, each supported by several subcategories, were identified, as presented in Table 3.

**Table 3:** Examples of main category, categories and subcategories

Subcategories	Categories	Main category
Being present Carefully observing and listening to resident’s needs Making autonomous decisions Building relationships	Caring about	The moral dimension of the provision of end-of-life care
Taking responsibility for the end-of-life care Share responsibility Inform and enable dialogue	Caring for	
Knowing what, why, how and when Knowledge and skills as preconditions for providing end-of-life care Being autonomous when making decisions.	Care giving	
Ongoing assessment of the end-of-life caring process and its outcomes Being aware not to provide maleficence care Balancing between too much and too little or not at all	Care receiving	
End-of-life care is a teamwork process The others and their influence on the end-of-life caring process Enabling leadership Caring within a ‘caring room’ Dignifying caring	Caring with	

Reporting the results was the last step in the analysis. To enhance the understanding of the study’s findings, the findings are presented with supporting excerpts from the participants.

### Rigour

In qualitative studies, trustworthiness is the main parameter for appraising the rigour of the study [45]. To enhance the trustworthiness of the study, four criteria—credibility, transferability, dependability and confirmability, as described by Lincoln and Guba [46]—were applied.

To support credibility, a detailed description of the sample and the sampling process was provided. Furthermore, the interview guide and the questions that the participants were asked during the interviews are made available to the readers. Moreover, although the data were collected from five individual interviews and one focus group, triangulation of two data collection methods allowed researchers to ensure that the study is based on diverse perceptions and experiences, strengthening the credibility and impact of the study’s findings [47].

Detailed information about the sample and setting supports the assessment of the transferability of the findings. In this way, the readers can recognise and evaluate whether the findings would be applicable to similar contexts with a similar sample. Quotes from the participants’ statements are given to support the findings. Each quote ends with a number representing the code that each nurse was given before conducting the interviews (i.e., Participant in interview 1, PI1 or participant 6 in focus group interview, P6FG).

To increase dependability, the same interview guide was used to ask all participants the same questions. Dependability was also increased by the researchers reading and analysing the interviews independently and then checking the consistency of the data analysis technique with each other and discussing the analytical process until a consensus was reached.

To enhance confirmability, excerpts from the participants’ statements were included when presenting the findings, thus verifying the concordance of findings with the raw data. This demonstrates that the data were not based on preconceived notions.

Trustworthiness was also supported by member checking, meaning that the researchers sent the participants the transcripts of the interviews immediately after data collection; then, the interviews were transcribed. The participants were asked to review the transcripts and check the accuracy of the data; hence, they had the opportunity to add, remove or clarify their statements. Only one participant answered this request, stating that the transcripts were accurate, and she did not have any further comments. Despite encountering a suboptimal response from participants, the authors remain confident in the trustworthiness of the study. Rich data, derived from a combination of individual and focus group interviews, yielded diverse and nuanced responses from participants, reinforcing the credibility of the findings.

Reflexivity is the researcher’s reflection on their position during the research process [48]. Both researchers have clinical experience in providing end-of-life care to nursing home residents. Therefore, it was critical to be aware of the impact that their clinical backgrounds might have on the research process from information seeking during the analysis of data and discussion of the findings. To avoid early interpretation of the data, the researchers were aware of their preunderstanding and tried to put it on hold. Both authors engaged in discussions regarding apprehensions and reflections, actively participating in the triangulation process throughout the study to prevent potential bias during data collection, analysis, and interpretation. The theoretical framework was brought in the end of the analysis process, which helped label the emerged findings.

### Findings

The analysis of the empirical data combined with an ethical reflection helped researchers to identify and understand the moral dimension of nurses’ experiences with end-of-life care provided to nursing home residents. During the analysis, an overarching category emerged– ‘The moral dimension of the provision of end-of life care’– describing nurses’ perceptions about how their professional autonomy influences the moral dimension of end-of-life care provided to nursing home residents. The participants agreed that end-of-life care is a care process that undergoes several phases, with each phase having its own ethical quality or its own element of care, here according to Tronto’s moral qualities [34]. In the following section, the findings are described using Tronto’s identified moral qualities for each of the five phases of the care process [32–35].

#### Caring about—being attentive

For the participants, being autonomous was perceived as a feature that increased their awareness of the resident’s caring needs during their last days of life. The participants agreed that the caring process involves paying attention, listening and recognising residents’ unspoken needs. Moreover, it implies nurses being able to make autonomous decisions when deciding which needs to care about at one particular moment.

The participants agreed that the core values of providing end-of-life care were to alleviate suffering, maintain dignity and provide comfort care. The participants perceived caring about as having sufficient knowledge, along with the experience and autonomy in practice, as well as providing comprehensive end-of-life care for residents. For the participants, caring about during the end-of-life process means them being present and dedicated. This implies nurses carefully observing, autonomously acting, and making decisions based on their judgements, and thus, they can decide and choose their course of action promptly based on resident’s condition or side effects. Moreover, caring about involved participants being attentive to perceiving the residents’ needs when the residents could no longer articulate themselves. The participants expressed their worries about resident’s bodily deterioration, leading them to lose their ability to express needs, as shown by the following quote:

There is not much communication when residents go into their last stage of life. Well… some of them are consciously until their death, but most are sedated; therefore, it is necessary to use your knowledge and experience to assess not only their needs for food and liquids or bodily hygiene, but also, we have to monitor their response to pain killers and other medication, and if it’s too much or too little, we need to do what’s needed to reduce or increase the medication and not let them suffer (PI3).

Some of the participants expressed that attentiveness to the residents’ care needs was a skill based on their clinical gaze developed during their careers. Other participants discussed that building a close relationship with the residents while they still could walk and talk was a precondition that helped them develop a clinical gaze, hence facilitating the nurses’ being attentive. Attentiveness allowed the participants to do what was needed when knowing the residents’ needs during the provision of end-of-life care. This may be interpreted as the moral or ethical quality of caring about during the end-of-life caring process, as demonstrated by the following statement:

We have time to know the resident before their health condition worsens… We previously knew *what* they wanted and *how* they wanted… their stay at nursing home gives us the opportunity to know their preferences and needs. Morally, we are obliged to provide the same quality of care they received when they could express themselves (PI4).

#### Caring for—taking responsibility

According to several participants, another phase within the end-of-life caring process was taking responsibility to care for. The participants agreed that monitoring the residents in their last days implies assuming responsibility. Assuming responsibility was perceived as an autonomous caring activity. They also discussed taking this responsibility seriously, which is a moral dimension of the end-of-life caring process and, ultimately, of the nursing profession. Usually, this responsibility was taken by a nurse, but it also involved other healthcare personnel or even next-of-kin. Among these responsibilities, the participants mentioned that the end-of-life caring process included not only caring for the resident’s physiological and psychosocial needs, but also assigning permanent healthcare personnel to continuously monitor the resident. Although the participants were aware that they share responsibilities for the caring process, ‘who does what…’, they ultimately had the overall responsibility for the whole end-of-life caring process.

Another responsibility included communication, which included listening, providing information, and supporting the residents’ next-of-kin. One of the participants expressed this as follows:

When I observe that the resident’s health worsens, I inform the next-of-kin and invite the spouse or the children to a meeting together with the responsible doctor and I, and we inform the next-of-kin what they might expect. The end-of-life care is not only about the resident and their last days, but also is to care for their next-of-kin to meet their needs and to overcome guilt feelings, anger or sadness.… (PI1).

Another way to care for patients was to deliberately increase opportunities to exercise autonomy during the caring process. For instance, the focus group participants discussed issues around advanced life support during the resident’s last days of life. Being prepared and having knowledge were the preconditions that gave them the authority to identify and make decisions about residents’ needs in here-and-now moments, hence exercising their autonomy. Some participants shared their experiences with controversies between next-of-kins’ and nurses’ assessments of what is the best care for the residents during their last days of life. Therefore, the importance of taking the initiative to discuss and clarify the resident’s needs and preferences was emphasised during the focus group interview, as shown in the following quote:

Some next-of-kins express wishes for advance life support and hospitalisation for their loved ones… and sometimes, to meet their needs, we try this, but the resident is suffering. The resident comes back to us after one or two days… To avoid this, clear guidelines, and a dialogue between the resident, their next-of-kin and us at the very beginning [when the resident enters the nursing home] is important… I think that minimalising the occurrence of difficult or conflictual situations and relieving the sufferance is care for both resident and their next-of-kin (P8FG).

#### Care giving—knowing what, why, how and when

During the interviews, the participants also discussed the caregiving process and provided concrete examples of what their caregiving encompassed. Spending extra time with the resident, choosing to be in the room and holding their hand to maintain physical contact was perceived as an autonomous caring act and a deliberate choice. One participant described this as follows:

For me, it is important that the dying person feels or hears that I am here with him or her… how he or she feels in these moments matters to me. I do it because I want to do it.… (PI5).

Other participants said that being autonomous when they actually provided caregiving to residents helped them make continuous assessments based on knowledge about *what*, *how*, *how much*, *when* and *why* to care. Knowledge and skills were decisive factors in providing competent care and making autonomous decisions during the caregiving phase; hence, competence was perceived as a moral dimension of caregiving. One of the participants said the following:

Caregiving at end-of-life is not only about giving morphine according to the doctor’s prescription… it involves all the judgements you have to make, all the skills you have… from preventing the occurrence of bedsores to knowing when to stop feeding but preventing thirst… think about all this knowledge and experience you must have to be able to make autonomous bedside judgements about *when*, *why* and so on.… (PI2).

Care giving at the end-of-life was described as all the necessary activities a nurse does to provide comfort and compassionate care to a dying resident. Among these activities, providing fundamental care and keeping residents comfortable and free of pain were seen as parts of the caregiving process. Moreover, adequate pain relief and symptom management were described as the moral dimension of care giving at this stage of end-of-life care, as one of the participants from the focus group interview said:

You cannot be passive when you see that the resident is suffering. I cannot go home and think that I should have done one or the other. It is against the nurses’ code of ethics and my personal moral and ethical principles. You have to act… I have to do what is needed… first thing first… pain relief and then personal hygiene! (P9FG)

Some of the participants mentioned some challenges they encountered during the care giving process. They said that care giving implies also standing in demanding situations. The lack of healthcare personnel with necessary knowledge or formal palliative care education or handling ethical dilemmas was seen as demanding situations that influenced the provision of care giving. Most of the participants felt that they were alone during the decision-making processes, which increased their awareness of their professional autonomy:

Sometimes, during weekends or evenings, I am the only nurse among the healthcare staff, and I have an overall responsibility for all nursing home residents. I have to prioritise who gets my attention and who needs me the most. Things can happen, regardless of whether it is Friday evening or weekend. I have to make a decision and do what is needed: to be with the dying resident and to support his or her next-of-kin in that moment. (PI5)

#### Care receiving—assessing caregiving

Several participants stated that, during the care-giving process, it was important to assess how the resident receive the care provided at the end-of-life stage. This was possible by monitoring the resident’s state of being but to also assess the outcomes of their care giving activities. They also reflected on their assessments and how they subsequently dealt with those assessments.

All the participants were confident in their knowledge and with their care giving at the end-of-life stage. They were aware that their care activities had consequences for the residents’ physiological and psychosocial needs. The assessment of the resident’s state of being was made by nurses listening, observing and interpreting resident’s response to care giving as signs of comfort or discomfort. One of the participants explained this as follows:

When providing personal care, if the resident presents any signs that can be interpreted as discomfort, I think that priority number one is me not causing more pain or suffering. However, I also understand that this person needs more pain killers, so I have to make sure that this person receives adequate medicine. (PI5)

Some participants also discussed the importance of assessing their care giving activities. They mentioned the importance of their assessments of the benefits of all care giving against the burden of all interventions and treatments. Their professional autonomy allowed them to make decisions about how to eschew care giving that was inappropriately and burdensome and choose the best comforting care for the resident. The participants stated that knowledge and experience were important in making such decisions, and their professional autonomy facilitated making choices of the best and less burdensome care giving. One of the participants said the following:

We have to assess whether the care giving provided meets the resident’s needs or not, whether the care comforts or perceives it as a burden and how the resident responds to this provision of care. (PI4)

During the interviews, some of the participants revealed a feeling of guilt when assessing that care giving altered the resident’s state of being, thus leading to new needs for care. They also discussed that the moral obligation and intention to relieve the suffering of the resident should override the foreseen but unintended harmful effects of care giving, including medication or other care interventions. One of the participants shared her experience as follows:

I still remember the attitude some of us had for a while ago… too much or too often morphine depresses the respiration and leads to death… I was struggling with feelings of guilt and even moral distress when I observed residents were still suffering because the medication they received had little or maybe no effect. I called the doctor and explained the situation… usually, the experienced doctors listen to us… and he [the doctor] prescribed more morphine.… (PI3).

Documentation of the response to care giving was also an issue discussed during the interviews. Some participants emphasised the importance of keeping detailed reports for a proper assessment of the care giving and medication and its outcomes. All reports were digitally written. Informal discussions between nurses and next-of-kin were also documented, especially when next-of-kin evaluated the care their loved ones received. The participants indicated that the more written information there was, the better. One participant acknowledged the following:

There is no such thing as ‘too much information’… being open about the morphine’s side effects and what to expect in the next hours or days is important for them [next-of-kin]. It helps them understand that end-of-life care is a process, not a quick fix procedure. (PI5)

#### Caring with—It is a teamwork process

During the interviews, most of the participants reflected upon the end-of-life caring process and its occurrence within the context of care in nursing home. The participants discussed that end-of-life care is not only about the responsibilities nurses have towards residents and their next-of-kin, but also the responsibilities of others who may influence the caring process. They perceived the caring process as an interplay between residents, next-of-kin, and themselves, along with how they relate to each other, which influences the caring process. However, as several participants asserted, this process did not occur in a vacuum: it occurred within an organisational context, which then influenced the caring process from the very beginning. One participant emphasised the importance of stable healthcare personnel within a caring organisation:

High staff turnover does not facilitate good end-of-life caregiving. Both residents and their next-of-kin need continuity and predictability in caring for and among healthcare personnel. They need somebody they know and trust… being exposed to new people every day increases their stress levels. (PI1)

Other participants discussed the importance of the leadership style and how the leader’s support influenced the culture of end-of-life care at the ward. The participants revealed that, within a caring context where their natural potential was enhanced through an enabling leadership style, they felt that they could provide competent and compassionate end-of-life care. One of the participants from the focus group stated that a positive leadership style supports nurses’ professional autonomy, thus helping them control the caring process, to have independence and to increase their ability to make clinical decisions and competent judgements regarding resident’s end-of-life care. One participant shared her experience as follows:

My leader gives me the freedom to make decisions when it comes to deciding what is best for the resident… She [the leader] enables me to be autonomous during the caring process, and this makes me aware of *what* and *how* to care.… (PI2).

The participants from the focus group interview also discussed how the nursing home’s caring culture influences care practice. They perceived the nursing home’s caring culture as positive, enabling good end-of-life care but also defective and an obstacle to care. They emphasised the importance of providing dignifying end-of-life care for residents. During the focus group interview, two of the participants engaged in a dialogue:

End-of-life care is providing care to the most vulnerable people, and it should be dignified… To do so, I have to provide care in a ‘caring room’ filled with dignity. (P7FG)

Although next-of-kin and I have different perspectives of what good end-of-life care might be, we care together, we are a caring team which ensures in our own way that the resident receives competent care.… Yes, you [P7] mentioned this ‘caring room’… maybe we should open the door more often into this room and invite next-of-kin. (P6FG)

## Discussion

The aim of the present study was to explore nurses’ perceptions of how their professional autonomy influences the moral dimension of end-of-life care provided to nursing home residents. In the following, we discuss these perceptions in relation to Tronto’s [32, 35] ethics of care framework and other supporting literature. To identify the moral dimension of these perceptions, we have related them to the moral qualities corresponding to each phase of the care process, as described by Tronto [33, 35].

In the first phase of the care process—caring about—the participants discussed the importance of being attentive to which type of care needs to be provided, which is the moral quality of the first phase of care. Similar to findings from another study [49], findings from the present study revealed that some participants perceived autonomous practice as carrying out actions based on their decisions. *Caring about* entails detecting the resident’s needs, hence obliging nurses to ‘do something’ [50]. This particular skill was seen as an autonomous caring activity, that is, the nurses’ deliberate choice of putting on hold their self-interest and/or agenda and ‘a capacity genuinely to understand the perspective of the other in need’ [35] ^(p.34)^, here nursing home resident.

In Tronto’s view [33], nurses’ attentiveness contributes to building up a caring relationship with a patient. The findings from the current study reveal that nurses perceived the provision of competent and compassionate end-of-life care as a result of their clinical gaze developed through certain activities, attitudes and knowledge of the patient, and through mutual relationships between the residents, next-of-kin and them. These results are supported by findings from previous studies that emphasise the importance of the nurse’s past experiences with the resident [51] and the significance of developing a good relationship with the resident and their next-of-kin [8, 23, 52–54] to provide adequate care. Moreover, similar to findings from other studies [55, 56], the present study reveal that, to respond to the resident’s end-of-life care needs, nurses must bring not only their professional knowledge, clinical experience and ability to work autonomously but even ethical sensitivity. These findings enforce Gastman’s [50] view on caring, in which caring should respond to the patient’s care needs. This involves nurses having empathy, capacity of judgement and the ability to see what is required in a specific situation (here, end-of-life care), which, according to Gastmans [50], is inherent in the moral dimension of nursing practice.

The second phase of care—caring for—refers to nurses taking on the burden of meeting the needs identified in the first phase, that is, caring about. There was no ambiguity, and the participants had no doubts regarding who had the responsibility for the provision of end-of-life care to nursing home residents. The nurses’ responsibility was seen as a moral dimension of care. In line with Pursio et al.’s study [30], the present findings indicate that the freedom to make patient care decisions and work independently has a positive impact on the moral dimension of end-of-life care for nursing home residents. However, nurses’ work was not only about meeting residents’ needs, but also to create a safe milieu, a communicative space together with each other and with the resident’s next-of-kin, thus sharing power and control over the care process. Similar findings are displayed in an integrative literature review [53], demonstrating that a positive culture of collaborative and reciprocal relationships, a willingness to engage and become engaged and nurses communicating with intent to share and support rather than inform all lead to facilitating decision-making in nursing homes. According to Tronto [35], to facilitate end-of-life decision-making, nurses must take the initiative to allocate responsibilities; otherwise, the nurses withdraw themselves from their responsibility. By exercising their professional autonomy to assign responsibilities, nurses strive to mitigate the power imbalance among residents, their next-of-kin, and themselves, thereby preventing the occurrence of potential power struggles in their relationships [34]. This proactive approach helps prevent the emergence of end-of-life care dilemmas that could undermine the moral dimension of end-of-life care.

The third phase of care—care giving—requires, according to Tronto [35], the moral quality of nurses’ competence, meaning nurses directly engaging with care. The findings revealed that the nurses provided end-of-life care, and to do so, they needed to have competence, which implies the nurses having the knowledge, skills and values necessary to know what, why, how and when to provide end-of-life adequately. In addition, good end-of-life care requires the competence to individualise care—to provide competent care based on the resident’s physical, psychological, cultural and spiritual needs [57] while considering the resident’s context of care. Nurses’ competence is crucial for their autonomy; however, to effectively utilize their competence, nurses must be capable of assessing care needs and responding promptly [30]. Otherwise, delays in assessing residents’ care needs could undermine the moral dimension of end-of-life care. To provide individualised competent care, it is necessary that nurses make continuous assessments. As the findings reveal, the nurses were concerned with providing competent care, that is, adequate pain management. If the care provided was incompetent and led to more pain for the resident, the nurses perceived psychological distress—a state of being that resulted in response to a variety of moral events—leading to the nurses feeling anger, frustration, guilt, powerlessness and stress [58]. According to Tronto [34]^(p.17)^, ‘incompetent care is not only a technical problem, but a moral one’; however, as the findings reveal, the provision of competent care also depends on the nurses’ ability to prioritise decision-making when standing alone. Although nurses’ professional autonomy enabled them to make decisions and choose the right *what*, *how*, *how much*, *when*, and *why*, the lack of adequately educated healthcare personnel make the decision-making process a technical problem, which could weaken the moral dimension of end-of -life care.

The fourth phase—care receiving—involves the moral quality responsiveness. This means nurses being responsive to the reaction of the nursing home residents to end-of-life care process. As the findings have revealed, nursing home residents are vulnerable to nurses’ act of care or lack of care. According to Gastmans [59], care is a reciprocal practice that occurs within the framework of a relationship between the care provider (nurse) and care receiver (resident). The reciprocity consists of nurses assessing that the care provided actually meets the resident’s needs for pain management and other physiological and spiritual needs. The nurses had to make autonomous end-of-life care decisions to meet the resident’s needs. This involved the nurse’s attention to care giving to not be perceived as power abuse, which could have negative consequences for the moral dimension of end-of-life care provision.

According to Tronto [33], vulnerability may lead to unequal relationships where power abuse may occur. Nursing home residents are in a vulnerable position because they rely on nurses’ competence and ability to alleviate suffering and assess and reassess the residents’ responsiveness to pain management. To avoid an unequal relationship between resident and nurse, nurses must assess whether the care provided is competent or incompetent. Besides assessing and documenting the care provided and its outcomes, informal discussions between the resident’s next-of-kin and nurses were also assessed as important for next-of-kin perceiving a balanced power and equal position within the relationship. However, because each end-of-life act of care may alter the resident’s state of being, responsiveness requires more attentiveness [34]. Nurses must therefore meet the resident’s new needs for care with compassion and a commitment to maintaining the highest quality of life throughout the evolving stages of the resident’s end-of-life journey.

The final phase of care—caring with—requires that solidarity and trust are the foundation of all care giving to meet caring needs [35]. The moral quality of this caring phase is solidarity. The findings from the present study suggest that the nurses felt solidarity with both the residents and their leaders. The nurses felt that they were given the support and freedom to act autonomously when making decisions regarding end-of-life care, but similar to findings from a previous study [22], they also recognised the impact that organisational factors, such as leadership and care culture, may have on the justice and equality of the care provided when they prioritise care to whom needed it the most. Similar to findings from another study [49], participants in the present study described autonomy as the ability to make independent decisions and prioritise care for those who needed it most. However, according to Tronto [35], all nurses have a responsibility to help determine how care activities and responsibilities should be allocated. Residents, their next-of-kin and other healthcare personnel may have different views on how they may perceive appropriate, compassionate and dignified end-of-life care [20, 21].Therefore, it is important to have transparency in nurse–resident–next-of-kin relations if the element of power within the relationship should be replaced by trust. Otherwise, the nurses’ autonomy may negatively influence the moral dimension of end-of-life care provided to nursing home residents. By opening the door of the “caring room” and inviting next-of-kin to participate in the care process, nurses may contribute to a greater reflectiveness around what may constitute ‘good’ end-of-life care.

### Strengths and limitations

One of the strengths of the study is the use of Joan Tronto’s theory of the ethics of care [32, 34, 35] and its five phases and elements of care to discuss the study’s findings. This allows a deeper understanding of how nurses’ professional autonomy influences the moral dimension of end-of-life care provided to nursing home residents. Another strength lies in the utilisation of two distinct methods of data collection: individual and focus group interviews. These approaches provided diverse datasets that shed light on various aspects of how nurses’ professional autonomy impacts the moral dimension of end-of-life care. Furthermore, the inclusion of participants with varying work experiences from four nursing homes enhances the richness and depth of the data generated from the interviews, further strengthening the quality of the study. Member checking ensures that the researcher’s interpretations accurately reflect the participants’ experiences and perspectives, thereby enhancing the validity of the study. This practice can be considered one of the methodological strengths of the study.

The current study has also some limitations that need to be considered. First, a limitation may be related to the size of the participant sample. The sample consisted of only nine nurses, a number that may be seen as a limitation in data collection. To challenge this limitation, the researchers posed follow-up questions during the interviews, thus offering the participants the opportunity to provide rich descriptions of their experiences with end-of-life care. Even though the sample consisted of only nine nurses, these participants reflected on and described their everyday work experiences. The participants’ rich descriptions were evaluated as possessing sufficient information power [41], thereby enhancing the overall quality of dialogues during interviews– a notable strength.

Second, the findings are limited to these nine participants and their personal work experiences in four different Norwegian nursing homes. This means that the sample is small and context dependent, which may limit the transferability and generalisability of the findings.

A third limitation pertains to the potential influence of the chosen theoretical framework on researchers’ preunderstanding during data analysis. To avoid bias, the theoretical framework was introduced at the end of the data analyses and after the coding process was conducted. The theoretical framework contributed to situating the knowledge from the empirical data into theoretical knowledge and vice versa. However, to be certain about interpretations and knowing that the qualitative nature of the study cannot completely exclude the impact of the preunderstanding on the analysis of the data, both researchers were aware of their theoretical preunderstanding and tried not to make conclusions beforehand.

## Conclusion

The ethics of care framework provides opportunities for nurses to analyse their own caring activities during the provision of end-of-life care to nursing home residents. The exploration of the moral dimension of the provision of end-of-life care, utilising Tronto’s theory, revealed that moral qualities, such as attentiveness, responsibility, competence, responsiveness, and solidarity are influenced to a certain extent by nurses’ autonomy. What is crucial for the provision of competent end-of-life care is the nurses’ awareness of acting properly in accordance with the moral qualities to each of the phases of caring. Therefore, to provide competent end-of-life care nurses must be attentive to residents’ care needs, take on the responsibility for the care provided to ensure that residents’ needs are met, provide competent care based on knowledge, skills and values and assess how residents respond to the care provided. In other words, this is the basic nursing process in action, and this problem-solving approach is needed for the provision of competent end-of-life care.

## Data Availability

The data that support the findings of this study are not openly available due to reasons of sensitivity and are available from the corresponding author upon reasonable request. Data are located in controlled access data storage at Oslo Metropolitan University.

## Abbreviations

PI: Participant in interview [number of the individual interview
PFG: Participant [number] in Focus Group interview
